# CoFly-WeedDB: A UAV image dataset for weed detection and species identification

**DOI:** 10.1016/j.dib.2022.108575

**Published:** 2022-09-05

**Authors:** Marios Krestenitis, Emmanuel K. Raptis, Athanasios Ch. Kapoutsis, Konstantinos Ioannidis, Elias B. Kosmatopoulos, Stefanos Vrochidis, Ioannis Kompatsiaris

**Affiliations:** a Information Technologies Institute, The Centre for Research and Technology, Hellas, Thessaloniki 57001, Greece; b Department of Electrical and Computer Engineering, Democritus University of Thrace, Xanthi 67100, Greece

**Keywords:** Precision agriculture, UAV dataset, Weed detection, Deep convolutional neural networks, Semantic segmentation

## Abstract

The CoFly-WeedDB contains 201 RGB images (∼436 MB) from the attached camera of DJI Phantom Pro 4 from a cotton field in Larissa, Greece during the first stages of plant growth. The 1280 × 720 RGB images were collected while the Unmanned Aerial Vehicle (UAV) was performing a coverage mission over the field's area. During the designed mission, the camera angle was adjusted to –87°, vertically with the field. The flight altitude and speed of the UAV were equal to 5 m and 3 m/s, respectively, aiming to provide a close and clear view of the weed instances. All images have been annotated by expert agronomists using the LabelMe annotation tool, providing the exact boundaries of 3 types of common weeds in this type of crop, namely (i) Johnson grass, (ii) Field bindweed, and (iii) Purslane. The dataset can be used alone and in combination with other datasets to develop AI-based methodologies for automatic weed segmentation and classification purposes.

**Specifications Table**

**Table:** 

Subject	Computer Science
Specific subject area	Artificial Intelligence, Computer Vision and Pattern Recognition
Type of data	RGB images
How the data were acquired	Unmanned Aerial Vehicle - DJI Phantom 4 Pro *RGB Camera -* 1″ CMOS Effective pixels: 20 M with FOV 84° 8.8 mm/24 mm (35 mm format equivalent) f/2.8 - f/11 auto focus at 1 m - ∞
Data format	Raw Analyzed
Description of data collection	The RGB images were collected while the UAV was performing a coverage mission over the field's area. During the designed mission, the camera angle was adjusted to -87°, vertically with the field. The flight altitude and speed of the UAV were equal to 5 m and 3 m/s, respectively, aiming to provide a close and clear view of the weed instances. Utilized path planning platforms: Software: https://github.com/CoFly-Project/Waypoint-Trajectory-Planning Android DJI Adaptor: https://github.com/CoFly-Project/waypointmission
Data source location	Kileler, 415 00, Thessalian Plain, Larissa, Greece Latitude and longitude from the agricultural field: [39.54164,22.64298 39.54032,22.64436 39.54183,22.64687 39.54305,22.64542]
Data accessibility	Repository name: CoFly-WeedDB Direct URL to data: https://zenodo.org/record/6697343#.YrQpwHhByV4 DOI:10.5281/zenodo.6697343 Database description: https://github.com/CoFly-Project/CoFly-WeedDB/blob/main/README.md

## Value of the Data

• Presented data is a significant addition to the field of weed datasets, where the currently available are limited, especially in case of examining species of southern Europe.
• Data are valuable for computer scientists and electrical engineers conducting research or developing tools focused on precision agriculture.
• Collected data can be employed to train and evaluate AI-based methods for weed detection.
• Collected data can be utilized in general for computer vision approaches for object segmentation and counting, image analysis, etc.

## 1 Data Description

**Fig. 1:**
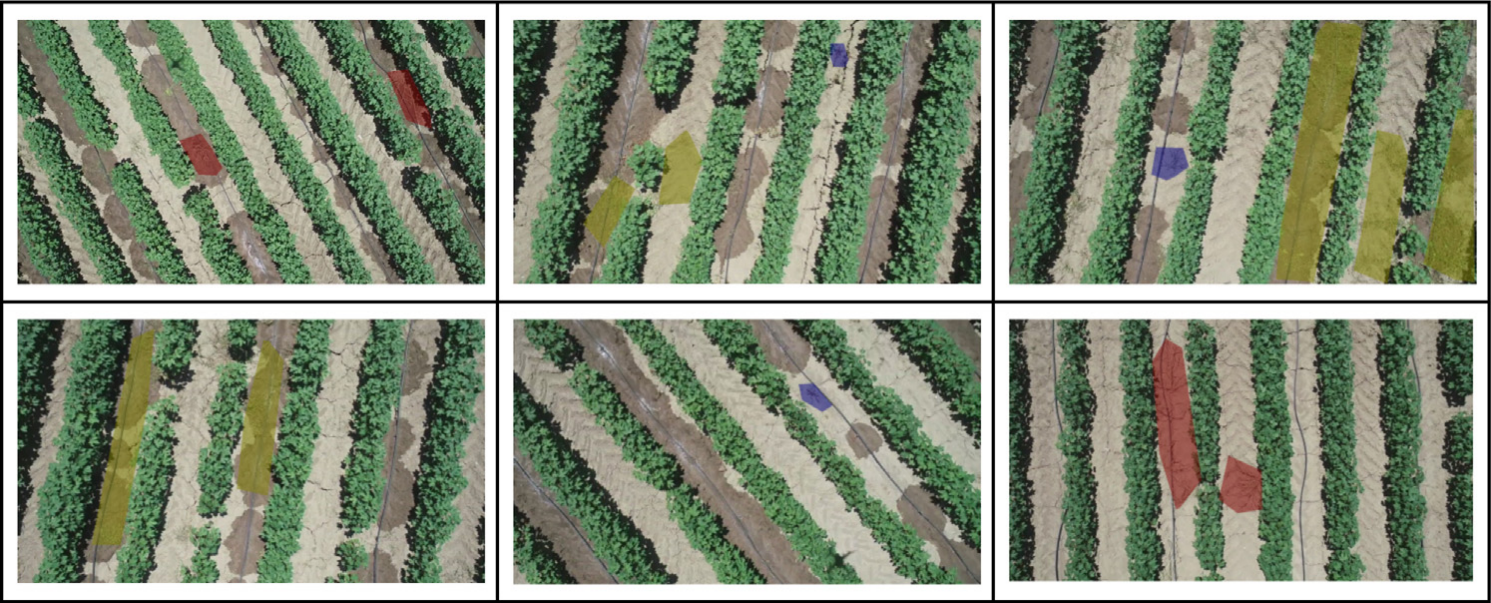
Sample images of the deployed dataset, where the annotation mask is overlaid over the captured RGB image for illustration purposes. Johnson grass is highlighted with red, purslane with blue and field bindweed with yellow color.

In this paper, a custom-built dataset is provided, oriented for precision agriculture operations. More specifically, data were collected during a UAV flight mission over a cotton field, leading to a set of 201 RGB images. Weed instances depicted in the acquired images were annotated accordingly by field experts, forming four classes, namely Johnson grass, Purslane, Field bindweed and Background. In Fig. 1 sample images from the developed dataset are provided, while in Table 1 a statistical analysis of the dataset classes is presented. The provided data can be utilized to train and evaluate weed detection methods, especially those based on semantic segmentation approaches.

**Table 1:** Statistical analysis presenting the number of pixels and instances per class contained in the custom-built dataset. As instance is mentioned a group of neighbour pixels that belong in the same class.

Class	Pixels (106)	Instances
Background	175	-
Johnson grass	1.44	77
Purslane	0.27	21
Field bindweed	7.56	286

## 2 Experimental Design, Materials and Methods

### 2.1 Dataset Acquisition

**Fig. 2:**
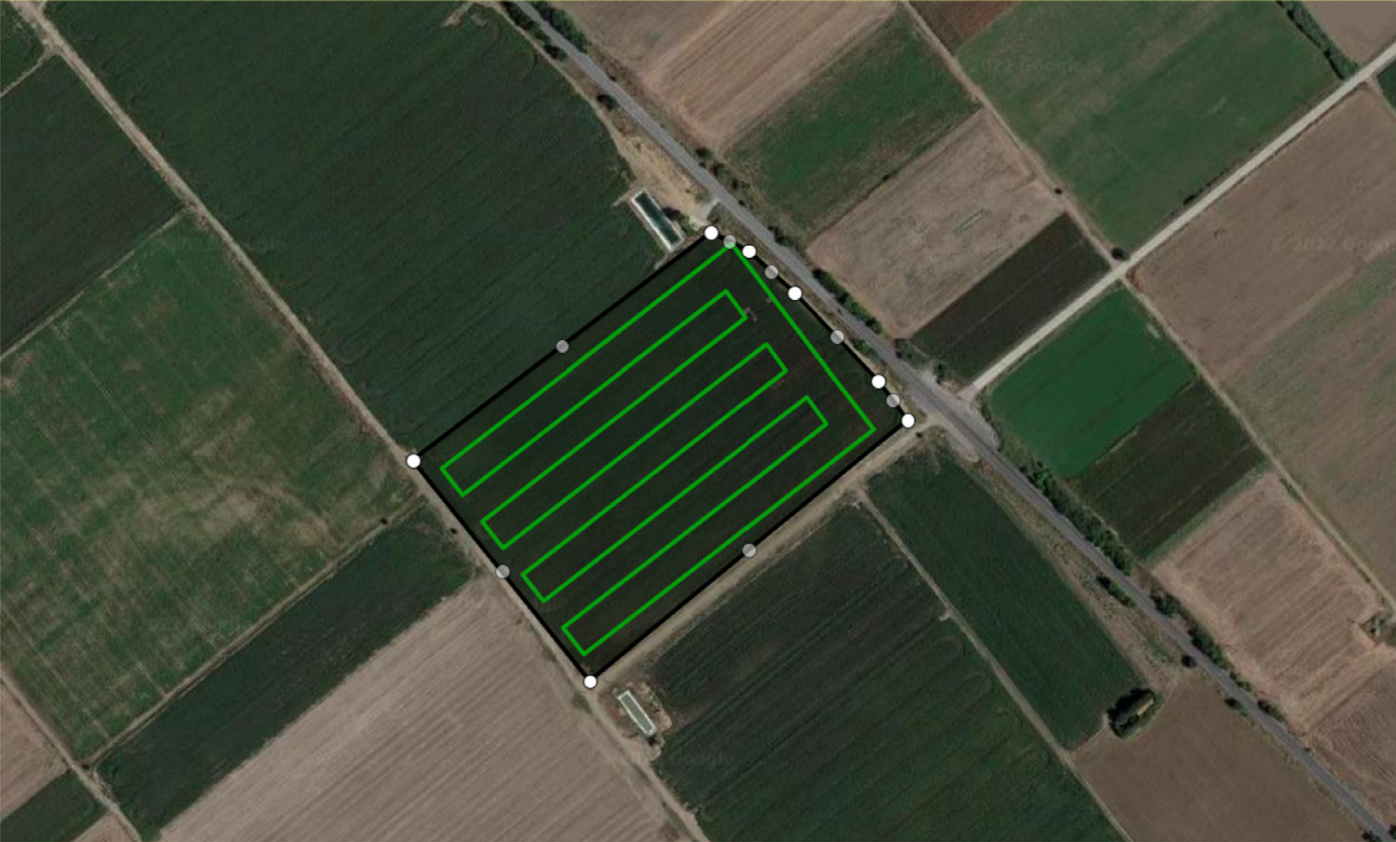
Top view of the data source location. The black lines indicate the boundaries of the cotton field while the green lines illustrate the UAV's coverage path for precision-based crop monitoring.

For the data collection, an autonomous UAV-based navigation scheme (Fig. 2) was deployed to cover the agricultural field completely, ensuring maximum possible efficiency in the mission while taking into consideration any constraints that could affect the objective (geometry shape of the field) and the integrity of the UAV (No-fly zones/obstacles, battery limitations, etc.). The autopilot system is based on a Coverage Path Planning problem [1], and it utilizes a Spanning Tree algorithm [2] to provide safe and efficient paths. The deployed UAV coverage path planning software is publicly available and can be found at [3].

For the image acquisition, a single commercial UAV (DJI Phantom 4 Pro) was used equipped with a visible camera sensor capturing light in red, green, and blue bands. For the mission control and execution of the extracted waypoints from the auto-navigation algorithm to the UAV, a custom and user-friendly interface based on the UAV's handling capabilities have been implemented while offering a simplified form of on-site commands [4]. Specifically, the android application (hosted on a Xiaomi Mi Max 2) is used as a data transceiver between the automatic navigation algorithm and the flight control system, allowing the UAV to follow the optimal path in order to cover an agricultural field and collect data. Finally, a portable 4G router (tp-link M7350) was used to handle wirelessly the connectivity and message routing between the smartphone and the path planning interface.

### 2.2 Collected Data Analysis

The deployed data collection mission led to a set of 201 RGB images of size 1280 × 720 pixels. Acquired images depict cotton crop lines, where different types of weeds interfere amongst the crop plants. The collected dataset was manually annotated by field-experts capable of confidently distinguishing weeds from cotton plants. Towards this direction, LabelMe [5] annotation tool was utilized to label depicted weed instances with polygonal annotations. Thus, every weed instance is labeled in detail with a polygon outline. The specific annotation approach was preferred to other approaches (e.g., bounding box), aiming to create a fine-grained annotated dataset capable of being utilized for semantic segmentation tasks, where crucial information regarding the shape and the location of the detected objects can be provided. Considering the dataset classes, three different classes were defined, namely Johnson grass, Purslane and Field bindweed, where different types of weeds are enclosed in each class accordingly, while the rest of the image is labeled as Background. In Fig. 1, a set of sample images from the developed dataset is presented, where it can be noticed the efficiency of the selected labeling approach to derive detailed and tailored to the depicted objects annotations. The deployed dataset is open-access and publicly available [6] to the research community.

As illustrated in Fig. 1, weed detection is a challenging task since weed clusters are captured in a wide variety of shapes, sizes, and types. Deploying a dataset that encloses the majority of existing cases is not an easy task and requires a heavy amount of workforce to collect data from a wide range of fields and annotate them accordingly. Furthermore, one should take into consideration that depicted weeds are usually capturing a small portion of the image, due to their natural size, especially in cases where UAV imagery is employed. Table 1 presents the number of pixels for background and weed classes, as well as the total number of weed instances enclosed in the developed dataset per class. Notice that due to the complex nature of the problem, the custom-built dataset is imbalanced, leading to a quite challenging detection task. Last but not least, the dataset, apart from the weed detection and identification, can be used for additional precision agriculture tasks, such as crop row detection [7], yield estimation [8], etc.

## Ethics Statement

Not applicable.

## CRediT authorship contribution statement

**Marios Krestenitis:** Conceptualization, Data curation, Writing – original draft, Visualization, Software. **Emmanuel K. Raptis:** Conceptualization, Writing – original draft, Software. **Athanasios Ch. Kapoutsis:** Conceptualization, Supervision, Project administration, Writing – review & editing. **Konstantinos Ioannidis:** Supervision, Project administration. **Elias B. Kosmatopoulos:** Resources, Supervision, Funding acquisition. **Stefanos Vrochidis:** Resources, Supervision, Funding acquisition. **Ioannis Kompatsiaris:** Resources, Funding acquisition.

## Declaration of Competing Interest

The authors declare that they have no known competing financial interests or personal relationships that could have appeared to influence the work reported in this paper.

## Data Availability

CoFly-WeedDB: A UAV image dataset for weed detection and species identification (Original data) (Zenobo).
